# Contradictions within the SDGs: are sin taxes for health improvement at odds with employment and economic growth in Zambia

**DOI:** 10.1186/s12992-019-0510-x

**Published:** 2019-12-18

**Authors:** Peter Hangoma, Gavin Surgey

**Affiliations:** 1 Department of Health Policy and Management, University of Zambia, Lusaka, Zambia; 2 Health Economics and HIV and AIDS Research Division (HEARD), University of KwaZulu-Natal, Durban, South Africa

**Keywords:** Sustainable development goals, Non-communicable diseases, Sugar, Sin taxes, Economic growth, Employment, Poverty

## Abstract

**Background:**

A recurring discussion in the literature relates to the possible contradictions among the Sustainable Development Goals (SDGs). The focus has been on economic goals, such as economic growth and goals related to climate change. We explore the possible contradictions that may arise between economic goals and health goals, specifically, the goal on Non-Communicable Diseases (NCDs) — SDG3.4. As a way to achieve SDG3.4, countries have been urged to introduce sin taxes, such as those on sugar. Yet others have argued that such taxes may affect employment (SDG 8.5), economic growth (SDG 8.1), and increase poverty (SDG1). However, there is limited or no reliable evidence, using actual experience, on the effect of sugar tax on health and economic outcomes. This makes it hard to assess the possible contradictions in SDGs that sugar taxes may generate.

**Main body:**

Using a conceptual framework on SDGs that views relationships among SDGs as either contradictory, reinforcing, or neutral, we carefully consider whether there are contradictions between SDG 3.4 on one hand and SDG 1, SDG 8.1, and SDG 8.5 on the other hand. We illustrate this using Zambia which recently introduced an equivalent 3% tax on non-alcoholic beverages, implicitly targeted at sugar-sweetened beverages (SSBs), given the stated goal of reducing NCDs. Concerns are that such a tax would be detrimental to the Zambia sugar value chain which contributes about 6% to GDP, in which case the achievement of SDG 3.4 (health) would be at odds with, or contradict, SDG 1, SDG 8.1, and SDG 8.5 (poverty eradication, economic growth, and creation of employment). We discuss that the existence of contradictions depend on a number of contextual factors, which allows us to make two conclusions about sugar taxation in Zambia. First, the current tax rate of 3% is likely neutral (no contradictions or reinforcing relationships) because it is too low to have any health or employment effects. However, the revenue raised can be reinvested to improve livelihoods. Secondly, the tax rate should be increased but care has to be exercised to ensure that the rate is not too high to generate contradictions. There will be need to carefully assess important parameters such as elasticities and explore alternative economic livelihoods.

**Conclusion:**

Without paying due consideration to important contextual factors, Zambia and many LMIC risk experiencing contradictions among SDGs.

## Background

### Introduction

In September 2015, world leaders gathered at the United National (UN) general assembly to agree on the 2030 agenda for sustainable development, the Sustainable Development Goals (SDGs). Unlike their predecessor, the Millennium Development Goals (MDGs), the SDGs were more comprehensive with 17 goals and 169 related targets spanning economic, social, and environment aspects. Though focused on low- and middle-income countries (LMIC), the SDGs also addressed industrialized countries calling them to reorganize their economic, social, and environmental order to prevent environmental degradation, including through sustainable production and consumption, so that the earth can support the needs of the present and future generations. Perhaps SDG 1—end poverty— is the backbone of the SDGs, which is also highlighted in the preamble: “eradicating poverty in all its forms and dimensions, including extreme poverty, is the greatest global challenge and an indispensable requirement for sustainable development.” A survey of experts also put ending poverty as the most important goal, only behind reducing inequality [1]. Poverty in this context is defined as living on less than $1.25 per day. Overcoming poverty may entail creating employment opportunities (SDG8.5) for all people in order to grow their incomes beyond $1.25 per day. The pursuit of other goals must therefore be assessed on how they related to SDG1, SDG8.5, and other economic goals.

There have been debates on the internal consistency of the SDGs [2], implying that most goals are interrelated and attempting to achieve one may result in another goals being negatively affected [3]. The most prominent has been the debate focused on the contradiction between the goal on economic growth (SDG8.1) and climate action (SDG 13) arguing that growth is not sustainable because it leads to environmental degradation [4]. Although others have highlighted the inconsistency between economic goals and social goals [2], we did not find any literature that has illustrated the possible contradiction between economic goals on poverty reduction (SDG1), economic growth (SDG8.1) as well as job creation (SDG 8.5) with the goal on health (SDG 3). Examining possible contradictions/trade-offs and the factors that may strengthen or dampening the trade-offs may help in making policymakers aware that in their quest to achieve the SDGs, they need to carefully assess possible interventions and courses of action and use those that may lead to contradictions among the goals. We explore possible contradictions between the economic goals and the health goal, and explore the factors that may make these contradictions more or less likely. We focus specifically on the target on NCDs (SDG 3.4), and one commonly proposed interventions, namely, tax of sugar-sweetened beverages (SSBs).

People today have better food, clothing, education, housing, health, and they live longer than their predecessors in the past two centuries [5]. These levels of prosperity have been partly driven by high levels of economic growth and innovation. Regions that have registered sustained improvement in economic conditions, e.g., economic growth, poverty reduction, and employment creation, have managed to reduce or eliminate many preventable deaths due to infectious diseases and birth complications. However, improved economic conditions have also contributed to a rise in lifestyle diseases related to obesity such as diabetes, cardiovascular diseases, and other non-communicable diseases (NCDs). These diseases have been partly attributed to excessive sugar consumption and tobacco use, with many, including the World Health Organization (WHO), calling for the imposition of taxes to discourage their consumption [6]. There are also calls for other economic measures to discourage their production.

Hence, these calls feed directly to SDG 3.4, which aims to reduce NCD mortality by one-third by 2030. This commitment was adopted in the 2018 political declaration on NCDs at the UN High Level Meeting in September 2018 (resolution: A/73/L.2) and included commitments to also scale up funding and responses to treat and prevent NCDs [7]. As of November 2018, 43 out of 194 WHO member states reported that they had implemented sugar-sweetened taxes as a way of curbing the increasing burden of NCDs with a number of LMIC following slowly [8].

However, the realities are that economic livelihoods are limited in most LMIC and industries such as those involved in sugar production and processing provide employment to a large share of the population, keeping them out of poverty. At the same time, these industries contribute significantly to economic growth. In high-income countries, as well as other LMIC, livelihood may not depend much on the sugar value chain. Hence, whether a sugar tax aimed at reducing NCDs, and thus achieving SDG 3.4, discourages production and consumption to the extent that the achievement of goals on ending poverty-SDG 1, increasing economic growth-SDG8.1, and providing decent work-SDG 8.5 are negatively affected depends on a number of contextual factors. Contradictions arise when a reductionist approach in implementation is taken to address individual goals rather than adopting systems thinking taking account of the context [9].

We draw special attention on Zambia, which recently introduced an equivalent 3% tax on non-alcoholic beverages with the stated goal of reducing NCDs [10], by reducing the consumption of non-alcoholic beverages [11]. This is consistent with international consensus that the primary objective of a sugar tax is to curb harm from sugar intake and fiscal policy is necessary to prevent noncommunicable diseases [12].

Examining the possible contradictions between health and economic goals would have required synthesizing the current evidence. However, the evidence on the impact of sugar taxes on health and on economic outcomes is weak, and mostly based on simulations, rather than actual experience [13]. While studies that use simulations report that sugar taxes improve health, those using actual experience do not find any health improvements resulting from sugar taxes [13]. There is no evidence of the employment effect in LMIC but the limited literature in advanced countries suggest little or no effect [14, 15]. Given the lack of reliable evidence, we use a modified conceptual framework on SDG to examine how sugar taxes may relate with health and other economic outcomes. We argue that there are no intrinsic inconsistencies/contradictions between health and economic SDGs. The contradictions are rather context dependent and hinge heavily on context factors as well as intervention/policy instruments used to achieve the health goal. Based on the identified context factor, we formulate hypotheses on the likely effects of SSB taxes on health, employment, growth, and poverty.

The rest of this paper proceeds as follows. In Section 1.2, we provided a context for Zambia, our focus country. We then provide a conceptual framework that can be used to assess relationships among SDGs in Section 1.3. The main text is in Section 2. Section 3 concludes.

### Context

Zambia is a very urbanized country with approximately 40% of the 16.8 million population residing in urban areas [16]. Despite being a lower middle income country, more than 54.4% of its population live below the national poverty line (23.4% urban and 76.6% rural area) and the country has some of the highest levels of income inequality in the region with a Gini coefficient of 55.6 [16, 17]. Health inequalities are also high [18]. Nonetheless, strong economic growth in Zambia, averaging 5% in the past 10 years and a large urban population share has seen rising incomes and changing lifestyles, especially in urban areas. Obesity and overweight prevalence has also been on the rise, with rates among women of child bearing age doubling in less than 15 years, from 12% in 2001 to 23% in 2014 [19]. In 2017, the overall prevalence of overweight/obesity was 24.2%, with women having a higher prevalence (32.5%) [20]. Obesity is a known risk factor for NCDs such as diabetes, hypertension and cardiovascular disease (CVDs) (WHO, 2016). Consistent with rising obesity, the incidence of NCDs in Zambia has been rising sharply with the total number of cases increasing by 56% between 2009 and 2011 [21]. NCDs accounted for almost a quarter all deaths in Zambia in 2017 [22].

Sugar sweetened beverage (SSBs) consumption has also been increasing. For example, in just 1 year, 2015 to 2016, the volume of SSB sold by Zambian Breweries, the largest distributers of soft drinks and clear beer in the country, grew by 4 % [23]. SSBs are becoming increasingly affordable for the general population of Zambia, with a 250 ml serving going for as low as K2 (about US$ 0.17). This makes it more likely for individuals to substitute healthier sources of calories or energy in favour of SSBs.

In line with the global call to introduce SSB taxes to curb NCDs, policy makers in the ministry of health have been attempting to move ahead with the WHO recommendation of taxing SSBs. They also argue that such a tax would raise revenue, which could be reinvested in the health sector. However, there is a complex political economy surrounding the introduction of an SSB tax. Policy makers in Ministries outside health are mainly concerned that the introduction of such a tax may lead to job losses across various stages of the SSB value chain, due a reduction in the demand for SSBs. This is a concern for Zambia as the sugar industry contributes more than 3% to GDP, 6% to total national exports and is directly responsible for more than 11,000 jobs [24].

Evidence for policy makers in Zambia on the likely effects on SSB taxes however has been lacking, with most of the literature focussed on high- and middle-income countries [13]. A modelling study at the request of the Ministry of Health showed that an excise tax could reduce consumption of SSBs, reduce obesity associated deaths, and increase revenue [25]. The study recommended that the revenue raised could be earmarked for health in light of the financing burden from NCDs.

In September 2018, the Finance Minister announced an equivalent 3% excise tax on ‘non-alcoholic beverages’. Although ‘non-alcoholic beverages’ is rather generic, the intention of the policy is to target sugar sweetened beverages as the stated goal of the tax is to reduce NCDs. By explicitly stating that the goal of tax is to reduce NCDs, there is a possible presumption that most of the affected products are sugar sweetened as the other two main non-alcoholic beverages—milk and water, may not matter much. This is because milk is tax exempt while for bottled water, the proportion of the population consuming it is very small (0.1%) [16]. Nonetheless, the 3% tax is far lower than the 25% tax recommendation by a modelling study [25]. Yet, this is a huge step in demonstrating Zambia’s commitment to curbing NCDs as key stakeholders initially indicated that the government was reluctant to adopt a sugar tax considering its possible employment and economic effects. Sugar accounts for Zambia’s most produced commodity with an average of 4.1 tons produced between 2012 and 2017 [26]. Zambia’s sugar industry accounts for 3–4% of the Gross Domestic Product (GDP), 6% of total national exports, and providing employment for around 11,000 workers [24]. The strategic importance of the sugar industry and the recent announcement of a sugar tax to curb NCDs makes it an interesting case study for possible trade-offs between economic and the health goals. While this is the case, it also worth mentioning that sickness imposes a huge burden on the economy at both the macro- and micro-level. At the micro-level, studies have shown that ill health may affect economic outcomes by reducing labour income, through reduction in productivity, and increasing medical spending [27]. At the macro-level, improved human capital and health budget savings arising from lower burden of NCDs may improve national income or Gross Domestic Product (GDP) [28, 29]. Whether a sugar tax will be improve economic outcomes through this channels, and hence lead to reinforcing effects, depends on its effect on health. The contradiction is a direct effect of a tax on economics outcomes. If the later effect is greater than the former, then the tax will generate contradictions between the health and economic goals. We formalize this discussion in the next section.

## Main text

### Conceptual framework

We use a slightly modified version of the SDG evaluation framework proposed by Singh, Cisneros-Montemayor [30]. While Singh, Cisneros-Montemayor et al. viewed the relationship between SDG goals as either intrinsically reinforcing, contradictory, or neutral, we posit that such relationships depend on the policy instrument used to achieve the SDG goal or target. For example, to achieve the target SDG 8.1.—economic growth of at least 7%—a government can adopt a capital intensive or labour intensive industrialization strategy. In this case, while this target will be contradicting SDG 8.4—the target of employment creation—if the strategy is capital intensive, it would reinforce it if the industrialization growth strategy is labour intensive.

**Fig. 1:**
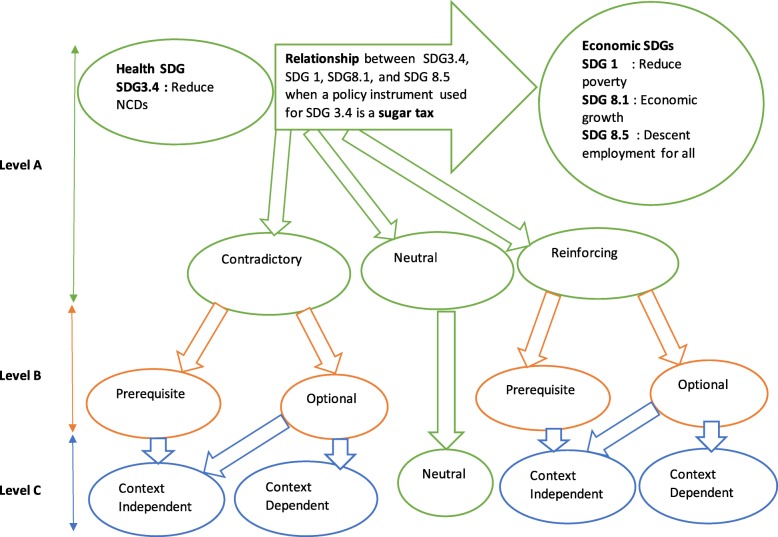
Modified Singh, Cisneros-Montemayor [30] SDG Evaluation Hierarchical framework that can be used to assess relationships among SDG targets. Level A checks the relationship (contradictory, neutral, or reinforcing) between two goals, Say one on reducing NCDs and the other on economic wellbeing, when a sugar tax is used as a policy instrument for SDG 3.4-NCD target. At Level B, Relationships are considered “prerequisite” or “optional” if achieving the NCD target using the sugar tax is needed to fulfil the economic SDG, or not, respectively. Level C shows the level of confidence in the established relationships. For relationships categorized as “context dependent” the contradictions or reinforcing relationships are dependent on the socio, economic, and cultural context

Formally, the SDG evaluation framework is hierarchical, with three layers (Fig. 1). At level A (green coded), the relationship between the SDG on reducing NCDs and the SDG on economic wellbeing is characterized as either contradictory, neutral, or reinforcing depending on the policy instrument Z (in this case sugar tax) being used to achieve SDG target X (reducing NCDs). In level B, the relationship is said to be prerequisite (optional) if reducing NCDs is required (not required) for the economic goals of reducing poverty, creating descent work are to be achieved. While reducing NCDs may lead to reinforcing relationships with, and hence improve, economic goals, many other factors, including education and reducing other disease, may improve economic outcomes. In our discussion thus, we rule out prerequisite relationships because SDG 3.4, reducing NCDs using any policy instrument, as it is not required to achieve economic growth, end poverty, or reduce unemployment.

Therefore, the goal of reducing NCDs can either be reinforcing-optional or contradictory optional to the goals of economic growth, poverty, and unemployment. It is reinforcing-optional because, as mentioned in the previous section, reducing NCDs could result in productivity gains as people become less sick. In addition, there may be savings in medical costs in the long term that could be used to invest in other productive sectors of the economy. However, there are cases when SDG 3.4 and the other economic targets could be contradictory-optional. This could be when the instrument used to bring down NCDs (e.g., sin taxes or sugar) leads to job losses or decreases job creation in this sector because of the reduction in production and consumption of the taxed product. In addition, if money that could have been used for poverty reduction strategies, such as providing clean water, are used to provide expensive treatment options for NCDs in a poor economy without consideration of cost effectiveness, the goal of reducing NCDs may contradict the other economic goals.

Importantly, these contradictory-optional and reinforcing-optional relationships can be either context independent or context dependent (level C). When two targets are contradictory or reinforcing irrespective of the context, they are said to be context independent, a special case that makes those specific SDGs intrinsically contradictory or reinforcing. At other times however, the relationship between two targets may be contradictory in certain contexts but not in other context. Similarly, they may be reinforcing in other context but not in other contexts.

## Discussion

Based on our conceptual framework, we hypothesize that the relationship between the goal on non-communicable diseases- SDG3.4 - on one hand and the poverty reduction goal (SDG1), goal on economic growth (SDG8.1) and goal on employment (SDG8.5) on the other hand is context driven. In other words, a sugar tax may lead to a contradiction between SDG3.4 and SDG1 as well as SDG8 in one country but not in another depending on the country specific factors. Crucially, even within the same country, modifying or paying more attention to context factors may yield more desired outcomes and avoid generating contradictions among the goals.

To be clear, a sugar tax has both benefits and costs. Benefits lead to reinforcing relationships among the SDGs while costs generate contradictions. The magnitude of costs relative to benefits is what ultimately determines whether SDG3.4 contracts SDG1, SDG8.1, and SDG8.5. There are three main economic benefits of a sugar tax: 1) direct tax revenue raised from the tax, which can be invested in other sectors of the economy to reduce poverty, create jobs, and enhance growth, 2) medical costs saved from treating and managing NCDs, which can be similarly reinvested, and 3) healthier workforce creating value from averted productivity losses. Similarly, reduction in productivity losses may improve household welfare, reduce the likelihood of poverty, and enhance the productivity capacity, and growth, of the economy.

The cost of a sugar tax is based on how much production and the related value chain decreases in response to reduced consumption. The decrease in production and value chain implies lower growth (SDG8.1). It may also lead to job losses (SDG8.5) as firms respond to reduced capacity. Job losses may increase the incidence of poverty (SDG1) if workers cannot transfer to a different industry.

The context factors that determine whether the costs outweigh the benefits include: 1) Size of the sugar value chain and availability of alternative economic opportunities; 2) Size of the SSB market, tax rate and how the market responds to the tax; and 3) How the revenues from the tax are utilized. We discuss in each these in return.

### Size of the sugar value chain and availability of alternative economic opportunities

In cases where the sugar value chain is a large share of the economy, taxing sugar may hurt employment opportunities, economic growth and ultimately increase poverty. Given that health is also determined by social economic factors, such a tax may hurt other health targets including those related to stunting, as households may not provide adequately for their children. These effects are likely to be a bigger concern in countries where there are fewer alternative economic opportunities to provide alternative livelihoods for those who have been rendered unemployed in the sugar value chain. In addition, taxing the sugar industry may also be a concern if alternate industries are unable to generate growth in the same way as the sugar value chain does, for example, through exportation and hence generating foreign exchange. Alternative economic opportunities are limited in Africa and the economic drivers are mostly reliant on mineral and oil resources. The growth in the agriculture sector is partly a result of an attempt to diversify and increase the value addition in other sectors beyond minerals. Agriculture is the key economic driver in most countries in the African region accounting for 50% of employment [31]. Sugar is one of highest economic value-added industries, outside of this there are few high value options, even within agriculture.

In Zambia, the sugar sector plays an important role and is one of the most successful non-traditional export crops. Zambia is one of the lowest cost producers of sugar in the world. It is ranked the world’s sixth lowest cost producer with an average cost of production in Zambia being US$169 per metric tonne compared to the world average of US$263 [32]. In 2016, the sugar sector accounted for approximately 3% of Zambia’s Gross Domestic Product (GDP) and 6% to total national exports in Zambia. Sugar is one of the main agricultural exports (in the top 5 commodities) with the sugar sector generating over US$45 million in gross export revenue annually (World Bank, 2007b). The country has historically been reliant of copper for export earnings and diversification has been a challenge due to limited alternative economic opportunities.

It is not only the size of the sugar value chain but also the limited alternative economic opportunities that make reducing NCDs through SSB taxes contradict the goals of employment, economic growth, and poverty. In the United States of America, SSB taxes have been associated with reduction in employment in the beverage sector [15], but overall employment may not be affected if there are employment opportunities in other sectors [14, 15]. In Hungary the decrease in SSB consumption had a negative effect on the economy [24]. Most countries countries that have introduced sugar taxes, e.g., South Africa, Brazil, Norway, and the UK, have wider economic opportunities and the share of sugar export earnings are not as important as they are for Zambia.

In the Zambian context, the sugar industry provided employment for around 11,000 workers in 2010, with a total of dependents exceeding 75,000 [24]. This has increased significantly in recently years, with just one producer, Zambia Sugar, estimated to support at least 11,474 jobs in 2016. Many more jobs are created by a number of outgrower schemes supporting sugar production. In addition, the secondary segment of the agriculture value chain also provides significant employment. The primary and agro processing industries employ more than 60% of Zambia’s total labour force. Sugar production is a high value agricultural industry with significant contribution to the manufacturing sector due to high value addition, diverse range of products and markets.

A tax that hurts production may affect jobs and people employed in the sugar value chain may have limited other economic opportunities. There would be challenges absorbing the workforce into other non-related sugar industry. The contradiction between economic goals and the goal of reducing NCDs using sugar taxes could be resolved by ensuring that alternative economic opportunities are available.

### Size of the SSB market and tax as well as how the market responds

In countries where the market for SSB is small, an SSB tax is unlikely to yield much health or revenue benefits. For Zambia, the proportion of individuals consuming SSBs was estimated at 14% [25]. The proportion is much higher in a number of countries that have introduced SSB, e.g., South Africa and the Philippines. It is for this reason that health and revenue benefits have been found to be modest in the Zambia [25], compared to the case of South Africa [33], or the Philippines [34].

The size of the tax on sugar will also determine the effects on NCDs and other economic aspects. The World Health Organization recommends threshold of 20% for an SSB tax (WHO, 2015). Yet it is important for each country to look at an appropriate tax rate that generates the highest gains in health and tax revenues while minimizing the negative impact on the economy. The tax rate should also be guided by the country specific responsiveness of consumption to the tax-or consumption elasticity of an SSB tax. If the elasticity is high, a small tax rate may have large consequences on consumption, production, and hence, health and other economic outcomes. High elasticity may also imply the presence of other substitutes so that when sugar beverages are taxed, consumer switch to other high calorie dense junk foods [35], hence diluting the health impact. If the industry generating alternatives is not of strategic importance—for example exporting as the sugar industry does-- it may not compensate the economic impact caused by lower sugar production, yet the health impact may have vanished or remained minimal. Thus, a case of high price elasticity of demand for SSB increases the likelihood of strong contradictions between the economic goals and the NCD target using a sugar tax. On the other hand, low elasticity may imply that only high tax rates can generate enough consumption changes that would have a discernible impact on NCDs. As a revenue measure, the tax would generate more tax revenues that could be reinvested in the health system, for example to promote healthy lifestyles. In the case of low price elasticity, the tax would be neutral or reinforcing. But whether or not this is realized depends on how authorities utilize the additional revenue.

The 3% tax on SSBs introduced in Zambia is far much lower than the 25% recommend by a modelling study that was conducted after consultation with officials from the Ministries of Health, Finance, and National Development Planning on the appropriate tax rate.

The modelling study found insignificant health and revenue impacts for tax rates lower than 20% in the case of Zambia. To yield the desired health and revenue benefits, the tax rate could be increased, but this has to be done after a careful analysis of the potential impact on employment and growth has been conducted. This will enable policy makers to set a tax rate that minimizes unintended consequences on employment and growth.

### How the revenues from the tax are utilized

Many countries are feeling increasing pressure to fund the growing health needs in their population. The rise of burden of disease from AIDS, TB and Malaria is a challenge, and the increasing rates of NCDs are costly to the health system. Many countries have also committed to providing Universal health care (UHC) in an attempt to make quality care accessible to all.

Zambia has recently passed their National Health Insurance (NHI) bill which is a mechanism to ensure a greater reach of health services and it aims to transform the Zambian health system. By comprehensively reforming how health is financed and how services are delivered, it reaffirms every citizen’s right to health. To implement National Health Insurance, extensive funding is required for its implementation. Even more important than the setup costs, are the effects of continued population growth and annual inflation, which will require consistent funding over the years to come. A sustainable source of funding is needed that assures lasting support of the health system that works for Zambia.

There is limited evidence on the amount of revenue raised from SSB taxation and how this is utilized. There is no doubt however that the benefits of a sugar tax will be greater if the sugar tax revenues are appropriately earmarked for health or other productive aspects of the economy. The danger is that if there is no prudent management of resources, the revenues raised may end up not yielding much value as they are mismanaged or misappropriated. Carefully earmarking revenue from other sin taxes to the health budget may yield positive results.

In the Philippines, taxes on tobacco and alcohol products generated enough revenue to triple the Department of Health’s budget since their implementation in 2012. It is found that sin taxes have consistently led to substantial health financing on top of direct health effects, with such benefits amplified among the most vulnerable populations [34].

The Indonesian central government is using funds from its regional tobacco excise tax to cover a budget deficit in the country’s health insurance program. The government’s strategy to cover the healthcare deficit is to take a portion of local administrations’ income from local cigarette tax and from tobacco excise revenue-sharing [36].

Earmarking funds raised from sin taxes is promising. However, in the case of Zambia, it is not a policy for any revenues to be earmarked or ringfenced for health (as is the case in neighbouring South Africa). So, if there are increased revenues raised from the SSB taxation, this would be allocated to the general tax pool and would not necessarily lead to increased funding for health. The SSB taxation would therefore not have any direct benefits on the health budget, but there could be overall benefits derived from the government having increased funds to allocate to the broader Government budget, if the funds are used prudently. One approach could be provision for subsidies to the agricultural sector to incentivize production of non-sugar related products or healthier products. This could stimulate production to compensate for the decrease in sugar-related agricultural produce.

## Conclusion

There are debates on whether SDGs are internally consistent; with others arguing that they are intrinsically contradictory so that pursuing one goal would negatively affect other goals. While most of this literature has focused on economic goals, such as economic growth and goals related to climate change, we explore the possible contradictions that may arise between economic goals and the goal on health, specifically, the target on NCDs. One of the instruments that has received global attention, and is recommended by WHO as a potent intervention for curbing NCDs is the sugar tax. Yet this tax raises important political economy dimensions. There are limited studies that have considered this political economy or looked at how reducing NCDs using sugar taxes may potentially contradict economic goals. This paper argues, with special reference to Zambia, that the NCDs and economic goals are not internally contradictory, but that these contradictions can arise if important contextual factors are not considered. These contextual factors determine the relative importance of benefits arising from an SSB tax—which give rise to reinforcing relationships among the SDGs—and the cost—which yields contradictions. We discussed three main contextual factors. First, we considered the size of the sugar value chain and availability of alternative economic opportunities. In the case of Zambia, the size of the value chain is high and there are few alternative economic opportunities. This context indicates a high cost (negative benefit) of implementing a tax in Zambia. Many recognize that an SSB tax would lead to lower consumption which results in lower production however, few consider if workers are able to switch employment to another sector and if investors are able to grow another sector. We need a method to measure cross elasticity of production (demand) whereby the change of demand in one sector leads to a shift of demand in another ‘productive’ sector, ensuring that jobs are not lost, and that the economy is still able to grow.

Second, we looked at the size of the SSB market, tax rate and how the market responds to the tax is quite small. While there is a positive benefit, it does not seem that the benefit gained would outweigh the negative benefits from alternative economic opportunities in section one. Third and finally, how the revenues from the tax are utilized are uncertain. The Ministry of Finance in Zambia does not earmark revenues raised from sin taxes, so this would not necessarily lead to increased budgets within the health sector.

In the case of Zambia, we conclude that current tax rate of 3% may not lead to contradictions between the NCD and economic goals. It could be increased to level that has been carefully considered, through evidence, that it minimises negative economic impacts. This is important because tackling NCDs through an SSB tax may be both reinforcing-optional and contradictory-optional so that which of these two forces dominates is ultimately determined by how the determined tax rate consider the important contextual factors we have identified. The biggest concern for Zambia that may make the relationship more contradictory is the limited alternative economic opportunities. This illustrates that an economy should never be highly reliant on a single or handful of sectors for productivity. A well-diversified economy gives workers and investors a greater opportunity to shift into other sectors should there be a decline in one sector, be it sugar, tobacco or oil.

In a nutshell, it is important to realize that development losses may occur if one SDG is pursued with looking at its interrelation with other goals. There is need to take a systems thinking approach. If a reductionist approach is taken and no attention is paid to contextual factors, health gains may entirely be offset by the economic losses from jobs, and economic growth. We may save funds from improved health, and even raise some funds from increased taxes however, if the economy becomes less productive there is less money to collect and more without jobs, which could pose a risk to the health of the people in turn.

The SDG framework is a useful reference for development. However, country specific evidence is necessary for decision making in charging how to each target is pursued.

## Data Availability

This article used available literature, and no data.
